# The impact of single versus double blastocyst transfer on pregnancy outcomes: A prospective, randomized control trial

**Published:** 2017-12

**Authors:** OM Abuzeid, J Deanna, A Abdelaziz, SK Joseph, YM Abuzeid, WH Salem, M Ashraf, MI Abuzeid

**Affiliations:** Department of OB/GYN, Hurley Medical Center, Michigan State University College of Human Medicine, Flint Campus, Two Hurley Plaza, Ste 101, Flint, MI 48503, USA; Department of OB/GYN, Genesys Regional Medical Center, One Genesys Parkway, Grand Blanc, MI 48439; Department of OB/GYN, Marian Regional Medical Center, 1400 E Church Street, Santa Maria, CA 93454, USA; IVF Michigan Rochester Hills & Flint, 3950 S Rochester Hills, Ste 2300, Rochester Hills, MI 48307, USA; University of Southern California, 020 Zonal Ave, IRD Room 533, Los Angeles, CA 90033, USA; Division of Reproductive Endocrinology and Infertility, Hurley Medical Center, Michigan State University College of Human Medicine, Flint Campus, Two Hurley Plaza, Ste 209, Flint, MI 48503, USA

**Keywords:** blastocyst transfer, single blastocyst transfer, double blastocyst transfer, IVF, ICSI, pregnancy outcome, Randomized Control Trial, RCT

## Abstract

**Objective:**

To determine if elective single blastocyst transfer (e-SBT) compromises pregnancy outcomes compared to double blastocyst transfer (DBT) in patients with favorable reproductive potential.

**Methods:**

This Randomized Control Trial included 50 patients with SBT (Group 1) and 50 patients with DBT (Group 2). All women were <35 years and had favorable reproductive potential. Randomization criterion was two good quality blastocysts on day 5. Patients who did not get pregnant or who miscarried underwent subsequent frozen cycles with transfer of two blastocysts (if available) in both groups.

**Results:**

No significant difference was observed in the majority of the demographic data, infertility etiology, ovarian stimulation characteristics and embryology data between the two groups. There was a significantly lower clinical pregnancy (61.2% vs 80.0%), and delivery (49.0% vs 70.0%) rates, but no difference in implantation (59.2% vs 54.0%), miscarriage, or ectopic pregnancy rates between Group 1 and Group 2, respectively. There was a significantly higher multiple pregnancy rate in Group 2 (35.0%) compared to Group 1 (0%) [P=0.000]. When fresh and first frozen cycles were combined, there was a significantly lower cumulative clinical pregnancy (77.6% vs 96.0%, P=0.007) and delivery (65.3% vs 86.0%, P=0.016) rates in Group 1 compared to Group 2 respectively.

**Conclusions:**

In patients with favorable reproductive potential, although e-SBT appears to reduce clinical pregnancy and live-birth rates, excellent pregnancy outcomes are achieved. Clinicians must weigh the benefits of DBT against the risk associated with multiple pregnancies in each specific patient before determining the number of blastocysts to be transferred.

## Introduction

Multiple pregnancies and its consequence is one of the complications of gonadotropin treatments and in-vitro fertilization-embryo transfer (IVF-ET). In this day and age, the success of IVF-ET is measured by the delivery of a single healthy baby. However, to maintain an acceptable pregnancy rate, two or more embryos are transferred by many units and this increases the risk of multiple pregnancies. Multiple pregnancies result in adverse outcomes such as higher risks of prematurity, low or very low birth weight, intrauterine growth retardation, pregnancy- induced hypertension, and caesarean section (Spellacy et al., 1990; Seoud et al., 1992; Yokoyama et al., 1995). Also, twin pregnancies have a six-fold increased risk of perinatal mortality and morbidity and a fourfold increased risk of cerebral palsy compared to singleton pregnancies (Lieberman, 1998; Stromberg et al., 2002). Therefore, there is no doubt that elective single-embryo transfer (e-SET) is one of the most important steps of minimizing complications due to multiple pregnancies.

Single-embryo transfer can minimize the incidence of twin gestation; however, it may also decrease live birth rates. Initiated by European researchers, global efforts have been directed toward reduction of multiple gestations (Templeton and Morris, 1998). Over the years this idea evolved and resulted in the concept of e-SET (Vilska et al., 1999). This concept has achieved a wide range of popularity initially in Europe and more recently in the USA. The European IVF-Monitoring Consortium (2016) reported that in 2012 across Europe, 30% of all transfers were single embryos (Calhaz-Jorge et al., 2016). The same report indicated that twin deliveries after IVF/ICSI-ET are still close to 17% in Europe (Calhaz-Jorge et al., 2016). In the UK it was required for all IVF centers to reduce their multiple pregnancies to less than 10% (Ledger, 2009). In Belgium the rate of multiple pregnancies decreased from 25% to 11.9% in 2004 with the introduction of a new law that requires SET to be performed in the first cycle in all patients younger than 36 years (De Sutter, 2006). The most recent guidelines established by the Society of Assisted Reproductive Technology (2017) recommend that in favorable patients who are less than 35-year-old, a single blastocyst or single cleavage stage embryo is to be transferred (Practice Committee of Society for Assisted Reproductive Technology, 2017). All other patients in this age group should have < 2 embryos (cleavage-stage or blastocyst) transferred. The Practice Committee of the Society for Assisted Reproductive Technology (SART) on elective single embryo transfer (2012) reported that in 2009 in the USA, e-SET accounted for approximately 10% of all transfers to patients less than 35 years old (Practice Committee of the Society for Assisted Reproductive Technology and Practice Committee of the American Society for Reproductive Medicine, 2012).

Several studies were done to compare e-SET with double embryo transfer (DET) (Pandian et al., 2013; Gremeau et al., 2012). Most of the randomized clinical studies that compared e-SET and DET examined cleavage stage embryos (day 2 or day 3) (Gerris et al., 1999; Martikainen et al., 2001; Lukassen et al., 2005; Van Montfoort et al., 2006; Moustafa et al., 2008). There is only one small randomized clinical trial that compared single blastocyst transfer (SBT) versus double blastocyst transfer (DBT) (Gardner et al., 2004). A Cochrane review by Pandian et al. (2013) showed that e-SET significantly reduces the risk of multiple pregnancies, but also decreases the chance of live birth in a fresh IVF cycle (Pandian et al., 2013). Subsequent replacement of a single frozen embryo achieves a live birth rate comparable with double embryo transfer (Pandian et al, 2013). Another systematic review and meta-analysis done by Gelbaya et al. (2010) to detect the likelihood of live birth and multiple birth after e-SET versus DET at the cleavage stage showed that e-SET of embryos at the cleavage stage reduces the likelihood of live birth by 38% and multiple birth by 94%. Evidence from randomized, controlled trials suggest that increasing the number of e-SET attempts (fresh and/or frozen) results in a cumulative live birth rate similar to that of DET (Gelbaya et al., 2010).

The objective of this pilot study is to determine if SBT compromises pregnancy outcomes compared to DBT in patients (<35 years) with favorable reproductive potential. We also compared the cumulative clinical pregnancy and delivery rates after fresh and the first frozen cycles between patients who started with SBT versus DBT and subsequently had a frozen-thawed cycle with DBT.

## Material and methods

Our study was a randomized clinical trial which was carried out between January 1st 2009 to October 31st 2013. It was approved by the Institutional Review Board at Hurley Medical Center, Flint, Michigan. Although the study was completed at the end of 2013, we had to wait for two and half years to obtain delivery outcomes of both fresh and frozen-thawed cycles. All patients presented with infertility during the period of the study, and were screened to determine if they fulfilled the inclusion criteria. Work up of infertility included: complete semen analysis, hysterosalpingogram, trans-vaginal ultrasound scan (2D and 3D) with saline infusion sono-infusion-hysterogram (SIH), hormonal profile including serum TSH, prolactin, day 3 follicle stimulating hormone (FSH) and luteinizing hormone (LH) levels and laparoscopy and hysteroscopy when indicated. Inclusion criteria was age < 35 years, day 3 FSH < 10 mIU/ml, no history of poor ovarian response, had no more than one previous IVF failure, no uterine cavity abnormalities and no contraindication to treatment medications or procedures. In patients who were found to have uterine factors such as submucus fibroid, endometrial polyps, uterine septum or significant arcuate uterine anomaly were not excluded from the study if the uterine abnormality was hysteroscopically corrected and post-operative SIH was normal.

Three hundred fifty-three patients qualified to participate in the study after fulfilling the inclusion criteria, but only 191 patients agreed to participate in the study after signing an informed consent. All patients received oral contraceptive pills (OCP) [Desogen, Merck & Co., Inc., North Wales, PA 19454, USA] 21-35 days in the preceding cycle. Controlled ovarian stimulation (COS) was achieved using mid luteal gonadotropin releasing hormone agonist (GnRH-a) protocol and mixed gonadotropins protocol starting on cycle day 2 or 3. Patients were monitored in a routine fashion during COS with trans-vaginal ultrasound scans (TVUS) for follicular size, endometrial thickness and serial measurements of serum estradiol (E2), serum progesterone (P4) and serum LH. Human chorionic gonadotropin (HCG), 5000 or 10,000 IU, was administered thirty- six hours before oocyte retrieval when 3 or more follicles were >16mm. Intracytoplasmic sperm injection (ICSI) procedure was used for all patients irrespective of the underlying etiology of infertility. Embryos were graded based on blastomere nuclear scoring on day 2 (Van Royen et al., 2003), and based on morphologic criteria on day 3 (Khan et al., 1991). Blastocysts were graded according to Gardner and Schoolcraft (1999) criteria. Patients with good quality embryos on day 3 (no. >4) were allowed to continue to participate in this study. Patients were randomized at the time of blastocyst transfer on day 5 by a computer-generated table to either transfer of one blastocyst (Group 1) or two blastocysts (Group 2); where odd numbers represented one group and even numbers represented the other group. This process was completed before the start of COS of the first patient in the study. The subject group assignment was blinded from the all study staff (nurse coordinator, nurses, embryologists, physicians) by placing the group assignment in sequentially numbered, sealed identical envelopes. The envelopes were prepared by a contracted research assistant who had no involvement with the recruitment, consent, assignment, or treatment of the subjects. One hundred patients fulfilled randomization criteria while 91 patients were not randomized. Once the patient was informed that she fulfilled the randomization criteria by having at least 2 good quality blastocysts (Grade 3 BB or better) the envelope was opened by the nurse coordinator or her designee and the finding was relayed to the couple and the staff. Fifty patients were allocated to Group 1 and another 50 patients were allocated to Group 2. In both Group 1 and Group 2 the best quality blastocysts were transferred. Cryopreservation of supernumerary blastocysts on days 5 or 6 was performed using vitrification method (Khan et al., 2012) for extra good quality blastocysts. Only blastocysts scoring Grade 3 BB or better by day 5 or day 6 were frozen. Trans-abdominal ultrasound- guided ET was performed on day 5. Patients who failed to conceive in the fresh cycle or those who miscarried after a fresh cycle underwent frozen thawed blastocyst transfer (FTBT) cycles. During subsequent FTBT cycles, DBT was performed in both groups (if 2 frozen-thawed blastocysts were available during their FTBT cycle).

Luteal phase support was the same in both groups starting on the second day after retrieval. Progesterone vaginal tablets T.I.D. (Endometrin 100 mg Vaginal Insert, Ferring Pharmaceuticals, Inc., Parsippany, NJ, 07054 USA) and estradiol B.I.D. (Estrace 2 mg, Warner Chilcott LLC, Rockaway, NJ 07866 USA) was utilized for the luteal phase support. If pregnancy occurred, luteal phase support was continued until six weeks gestation. Estrace was discontinued at six weeks gestation, while vaginal progesterone was continued until twelve weeks gestation. β HCG was measured twelve days after blastocyst transfer. Clinical pregnancy was defined as the presence of a gestational sac and a yolk sac on trans-vaginal 2D US scan. Miscarriage was defined as a clinical pregnancy that ended in pregnancy loss prior to twenty weeks gestation. Preterm birth rate was defined as any birth before 37 weeks gestation, while severe preterm birth was defined as any birth before 32 weeks gestation. During FTBT cycles, the transfer was performed during the natural cycle if the patient had regular ovulatory cycles, while in patients with irregular cycles the transfer was done during a medicated cycle with E2 and P4 (Le QV et al., 2017). The primary outcome measure was the reproductive outcome of the first fresh IVF cycle. The secondary outcome measure was the reproductive outcome of a combined fresh and FTBT cycle.

Statistical analysis was performed with independent Student’s T-test for comparison of continuous variables and Chi-squared analysis for comparison of categorical variables. In addition, multiple Binary logistical regression was used to assess the impact of group and significant demographic, ovarian stimulation characteristics and embryology data on clinical pregnancy rate during the fresh cycle. A P-value < 0.05 was considered statistically significant. SPSS version 23.0 (IBM, Armonk, New York, USA) was used.

## Results

**Figure 1:**
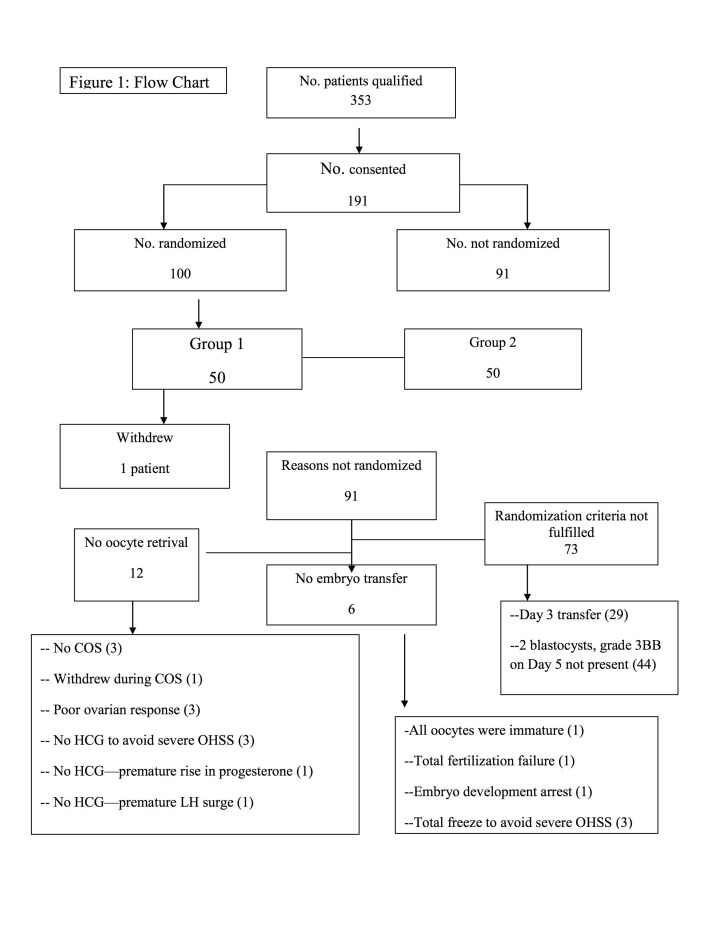
— Flow chart.

Figure1 illustrates a flow chart. Three hundred fifty three patients qualified to participate in the study after fulfilling the inclusion criteria. A total of 191 patients agreed to participate in the study and signed the necessary consent forms. One hundred patients fulfilled the randomization criteria on day 5 after retrieval. Fifty patients were randomized to Group 1 (SBT) and 50 patients were randomized to Group 2 (DBT). One patient in Group 1 decided to drop out of the study after she found out that she was randomized to Group 1 and asked to have DBT. This patient was excluded from the analysis. The remaining 49 patients in Group 1 received SBT. All 50 patients in Group 2 received DBT. Ninety-one patients were not randomized. In 12 patients oocyte retrieval was not performed. Three patients decided not to go with infertility treatment for social reasons and withdrew from the study before starting COS. One patient decided not to continue with infertility treatment for social reasons and withdrew from the study during COS. In 3 patients the cycle was cancelled during COS due to poor ovarian response. In 5 patients HCG was not administered and oocyte retrieval was cancelled; in 3 of these patients this was done to avoid severe ovarian hyperstimulation syndrome (OHSS); in 2 of these patients oocyte retrieval was cancelled; in 1 patient because of premature LH surge and in the other patient as a result of premature rise in progesterone (3.2 ng/ml) on planned day of HCG. In 6 patients no embryo transfer was performed; in 1 patient because all oocytes were immature; in 1 patient because of total fertilization failure; and in another patient because all embryos arrested at the zygote stage. In the remaining 3 patients embryo transfer was cancelled to avoid severe OHSS, and therefore total cryopreservation was performed. Seventy three patients were not randomized. Forty four patients did not fulfill the randomization criteria on day 5. In twenty nine patients embryo transfer was deemed necessary on day 3, as the number of cleavage stage embryos of good quality was less than four.

There was no significant difference in the demographic data or infertility etiology between the groups except for percentage of patients with endometriosis and percentage of patients with history of uterine factors (Table I). Table II shows no significant difference in the ovarian stimulation characteristics between the two groups except for percentage of patients that required coasting and percentage of patients who required 5000 IU of HCG as a trigger shot. Table III illustrates embryology data of the patients that were randomized. There was no significant difference in the majority of embryology parameters between the two groups except for significantly lower number of mature oocytes and significantly higher percentage of patients with excellent blastocysts transferred in Group 1 compared to Group 2. Table IV illustrates that there was a significantly lower clinical pregnancy (61.2% vs 80.0%, P=0.040) and delivery (49.0% vs 70.0%, P=0.033) rates in Group 1 compared to Group 2 respectively. Table 4 also illustrates that there was no significant difference in implantation (59.2% vs 54.0%), miscarriage (13.3% vs 10.0%), or ectopic (3.3% vs 2.5%) rates between Group 1 and Group 2 respectively. As expected, there was significantly higher incidence of multiple gestations (35.0% vs 0%, P=0.000) and higher percentage of multiple live birth (30.0% vs 0.0%, P=0.001) in Group 2 compared to Group 1 respectively (Table IV).

**Table I:** Demographic data.

	Group 1 (n=49)	Group 2 (n=50)	P-value
Age (yrs)	29.4 ± 3.3	30.6 ± 2.8	NS
BMI (Kg/m2)	26.7 ± 5.8	24.9 ± 6.6	NS
Duration of infertility (yrs)	2.6 ± 1.6	3.2 ± 2.4	NS
Day 3 FSH (mIU/ml)	6.1 ± 1.8	6.0 ± 1.7	NS
Day 3 LH (mIU/ml)	6.3 ± 5.1	6.0 ± 3.5	NS
% Primary Infertility	60.4%	66.7%	NS
% parity	23.3%	18.8%	NS
% Miscarriage	16.7%	12.5%	NS
% Male factor	55.1%	52.0%	NS
% Ovulatory disorder	34.7%	36.7%	NS
% Tubal factor	28.6%	18.0%	NS
% Endometriosis	26.5%	48.0%	0.027
% Unexplained	2.0%	4.0%	NS
% History of uterine factors*	30.6%	50.0 %	0.049

Any uterine factor such as sub-mucous fibroid, endometrial polyp or uterine septum was corrected via hysteroscopy once the pathology was found prior to IVF-ET treatment.

**Table II:** Ovarian stimulation characteristics.

	Group 1 (n=49)	Group 2 (n=50)	P-value
Baseline serum E2	35.1 ± 22.9	32.74 ± 13.0	NS
Baseline serum P4	0.3 ± 0.2	0.3 ± 0.2	NS
Baseline FSH (mIU/ml)	4.5 ± 4.0	4.6 ± 4.6	NS
Baseline LH (mIU/ml)	3.2 ± 2.9	3.4 ± 2.5	NS
Total dose of Gonadotropin	3131.8 ± 1790.5	2589.1 ± 773.4	NS
Total duration of treatment (days)	10.5 ± 1.6	10.1 ± 1.6	NS
E2 on HCG day	2589.1 ± 1075.2	3002.5 ± 1101.4	NS
P4 on HCGday	0.6 ± 0.3	0.7 ± 0.3	NS
LH on HCG day	3.0 ± 3.1	2.9 ± 1.9	NS
No. foll.> 16 cm on HCG day	7.8 ± 3.5	8.6 ± 4.2	NS
Endometrial thickness on HCG day	10.5 ± 2.5	10.9 ± 2.3	NS
% Coasted	17.8%	37.8%	0.034
% HCG dose of 5000 IU	2.0%	4.0%	NS
% History of uterine factors*	39.6%	62.5%	0.025

**Table III:** Embryology data.

		Group 1 (n=49)	Group 2 (n=50)	P-value
# Mature oocytes		10.7 ± 4.4	13.21 ± 6.4	0.034
#2PN		8.9 ± 3.3	10.3 ± 4.8	NS
# Day 3 embryos		8.9 ± 3.2	10.3 ± 4.4	NS
	# blastomeres >8	6.4 ± 3.0	7.4 ± 3.5	NS
	# blastomeres <8	2.5 ± 2.2	2.8 ± 2.3	NS
	# no fragments	6.4 ± 3.3	6.9 ± 4.4	NS
	# fragments	2.5 ± 1.9	3.4 ± 2.9	NS
# Day 5 blastocyst		6.3 ± 2.8	6.5 ± 3.4	NS
# Day 6 blastocyst		0.5 ± 1.3	1.0 ± 1.6	NS
Total no. blastocysts		6.2 ± 3.1	6.9 ± 3.3	NS
% blastocyst formation (from Day 3)		70.8%	66.8%	NS
# Day 5 frozen blastocyst		4.4 ± 2.7	3.7 ± 3.2	NS
# day 6 frozen blastocysts		1.0 ± 1.5	1.1 ± 1.5	NS
Total # frozen blastocysts		4.7 ± 2.7	3.9 ± 3.0	NS
Total # Excellent blastocysts		2.8 ± 1.2	3.2 ± 2.0	NS
Total # good blastocysts		3.5 ± 2.5	3.6 ± 2.0	NS
% patients with excellent blastocysts transferred		100.0%	82.0%	0.002
% Assisted hatching		10.6%	21.3%	NS

**Table IV:** Pregnancy outcomes pet ET.

	Group 1 (n=49)	Group 2 (n=50)	P-value
Implantation rate	59.2%	54.0%	NS
# Clinical preg. (%)	30 (61.2%)*	40 (80.0%)	0.040
# Delivered (%)	24 (49.0%)	35 (70.0%)	0.033
# Miscarriage (%)	4 (13.3%)	4 (10.0%)	NS
# Ectopic (%)	1 (3.3%)	1 (2.5%)	NS
# Multiple pregnancies (%)	0 (0%)	14 (35.0%)**	0.000

*1 patient had termination of pregnancy for anencephaly.

** 13 sets of twins and 1 set of triplets (one patient with twins had late second trimester miscarriage at 19 weeks gestation, 2 patients with twins had spontaneous reduction of one twin at 10 weeks and 6 weeks gestation).

**Figure 2:**
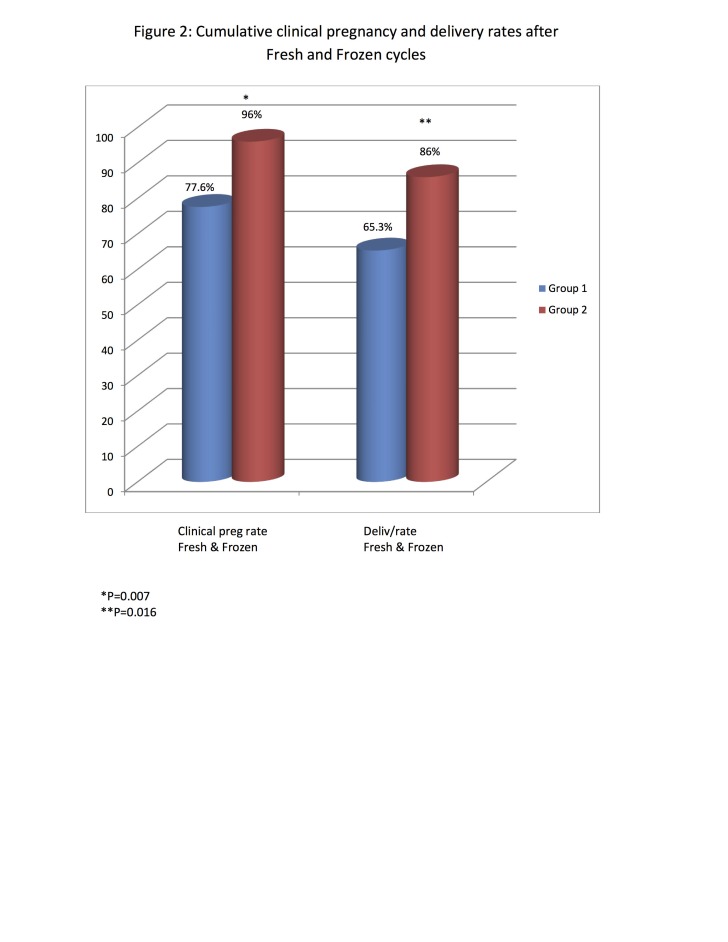
— Cumulative clinical pregnancy and delivery rates after Fresh and Frozen cycles.

There were 18 subsequent FTBT cycles in patients in Group 1 who had SBT in the fresh cycles. Sixteen patients (88.9%) had DBT while two patients had SBT (11.1%). Ten patients conceived (55.6%), nine delivered (50%) and one miscarried (10%). There were 12 subsequent FTBT cycles in Group 2 who had DBT in the fresh cycles. Ten patients (83.3%) had DBT while two patients had SBT (16.7%). Nine patients conceived (75%), seven delivered (58.3%), one miscarried and one had an ectopic pregnancy. Figure 2 illustrates that when fresh and the first frozen cycles were combined, there was a significantly lower cumulative clinical pregnancy (77.6% vs 96.0%, P=0.007) and delivery (65.3% vs 86.0%, P=0.016) rates in Group 1 compared to Group 2 respectively. There was a significantly lower cumulative multiple pregnancy rate (7.9% vs. 35.4%, P=0.003) and cumulative multiple live birth rate (7.9% vs 29.2%, P=0.014) in Group 1 compared to Group 2 respectively. During the fresh cycles there was no significant difference in the mean gestational age (37.3 + 4.0 vs 36.9 + 3.5), incidence of preterm birth (<37 weeks gestation) [20.8% vs 28.6%] and incidence of severe preterm birth (<32 weeks gestation) [8.3% vs 8.6%] between Group 1 and Group 2 respectively. When multiple Binary logistical regression was used to assess the impact of group (95% CI of 0.83 – 8.30) and significant demographic characteristics [percentage of patients who had endometriosis (95% CI of 0.19 – 1.62), history of uterine factors (95% CI of 0.88 – 7.30)], significant ovarian stimulation characteristics [percentage of patients who underwent coasting (95% CI of 0.19 – 2.60), or received HCG of 5000 IU as trigger shot (95% CI of 0.22 – 2.09)] and significant embryology data [# mature oocytes (95% CI of 0.88 – 1.06) and percentage of patients with excellent blastocysts transferred (95% CI of 0.04 – 3.88)] on clinical pregnancy rate during the fresh cycle it was found that there was no statistically significant effect.

During the fresh cycles there were 13 sets of twins and one set of triplets (one blastocyst resulted in monochorionic diamniotic twins and the other blastocyst resulted in monochorionic monoamniotic pregnancy) in Group 2. Of the 13 sets of twins, one patient had late second trimester miscarriage at 19 weeks gestation and 2 patients with twins had spontaneous reduction of one twin at 10 weeks and 6 weeks gestation. The patient with triplet delivered at 28 weeks gestation. The other 2 patients delivered at 33 weeks and 39 weeks respectively. The gestational age at which the remaining 10 patients delivered were as follows: One patient delivered at 28 weeks gestation, 2 patients delivered at 34 weeks gestation, 2 patients delivered at 35 weeks gestation, 1 patient delivered at 36 weeks gestation, 2 patients delivered at 37 weeks gestation and 2 patients delivered at 38 weeks gestation. Three singleton pregnancies in Group 1 ended in deliveries at 22, 31 and 33 weeks gestation, while one singleton pregnancy in Group 2 ended at 27 weeks gestation. It is worth of note, that the numbers of pregnancies and live births in the two groups were small as to make a meaningful statistical comparison. However, in our pilot study these admittedly small numbers suggest a poorer pregnancy outcome following double embryo transfer. During FTBT cycles there were 3 patients who had twin gestation in Group 1; they delivered at 36, 37, 38 weeks gestation. In addition, during FTBT cycles in Group 2 there were 3 patients who had twin gestation, and they delivered at 36, 37, 38 weeks gestation.

## Discussion

In recent years it is well acknowledged that a successful IVF treatment is measured by achieving a viable singleton delivery (Henman et al., 2005). The initial enthusiasm of avoiding multiple pregnancies after IVF-ET is now replaced by the desire to achieve a singleton live birth (Templeton and Morris JK, 1998; Vilska et al., 1999; Ryan et al., 2007; Styer et al., 2008; Gleicher et al., 2007). Elective SET/SBT is the only way to assure a singleton live birth after IVF-ET. This is now possible as a result of recent advances in both the clinical and laboratory aspects of assisted reproductive technology (Gardner et al., 2004). This is also in part due to better techniques of cryopreservation and thawing of the blastocyst (Henman et al., 2006). Many units are now advocating e-SBT in patients who are < 35 years old. Published data support the concept of using SBT instead of cleavage stage embryos whenever a single embryo transfer is planned (Gardner et al., 2004; Papanikolaou et al., 2006; Zech et al., 2007; Blake et al., 2007; Papanikolaou et al., 2008; Guerif et al., 2009). However, many groups are still transferring more than one embryo/blastocyst in patients with potential favorable outcome (<35 years old) (Gleicher and Barad, 2009).

The present study confirms the notion that in infertile patients less than 35 years, with potential favorable outcome, SBT can achieve very high clinical pregnancy rates, along with acceptable delivery rates. In addition, very high cumulative clinical pregnancy and delivery rates are to be expected after fresh and FTBT cycles are performed. In our study we did DBT in subsequent FTBT cycles in patients who failed to conceive or ended in miscarriage during the fresh cycle upon the patient’s request in both groups. This occurred in 88.9% of patients in Group 1 and 83.3% in Group 2 during their FTBT cycles. In patients who had SBT, there were no monozygotic twins during the fresh cycle, but the incidence of dizygotic twins during FTBT was 7.9%. Therefore, based on our data such patients should be counseled that e-SBT is associated with a very good reproductive potential. Such patients should be encouraged to have e-SBT, especially if they have extra blastocysts suitable for cryopreservation. Our data is in agreement with the original work of Gardner et al. (2004) who published the first randomized controlled trial comparing SBT to DBT, in which they achieved very high implantation (60%) and clinical pregnancy (60%) rates with SBT (Gardner et al., 2004). Our data and Gardner et al. (2004) data confirm the notion that clinical pregnancy rates are much higher than published data on e-SET at the cleavage embryo stage (Gardner et al., 2004). In addition, our data confirm the same notion regarding delivery rates. Other published data that compared SBT to single cleavage embryo (day 2 or day 3) transfer suggest better results with SBT (Papanikolaou et al., 2006; Zech et al., 2007; Blake et al., 2007; Papanikolaou et al., 2008; Guerif et al., 2009). Therefore, it is reasonable to suggest that if elective single embryo transfer is planned, it is better to do it at the blastocyst stage.

In contrast to Gardner et al. (2004) the present study is also showing that DBT is associated with significantly higher clinical pregnancy and delivery rates compared to SBT (Gardner et al., 2004). In addition, as would be expected, it was also associated with a significantly higher incidence of dizygotic twins (35.0%). Furthermore, our data shows that the cumulative clinical pregnancy and delivery rates after fresh DBT/FTBT cycles were significantly higher than when fresh SBT/FTBT using DBT was performed. As expected, twin pregnancy rate was also higher with DBT compared to SBT (35.4% vs 7.9%, P=0.003) respectively. Our data are not in agreement with Gardner et al. (2004) who reported no significant difference in clinical pregnancy rates between SBT and DBT (Gardner et al., 2004). In their study there was a tendency for higher pregnancy rate after DBT compared to SBT (76.0% vs 60.9% respectively), but the difference was not statistically significant most likely as a result of a small population size as pointed out by the authors (Gardner et al., 2004).

Forman et al. (2013) concluded that in women less than 42 years old, transferring one euploid embryo resulted in an ongoing pregnancy rate similar to transferring two untested embryos, while decreasing risk of multiple pregnancies. Their data highlight the role for pre-implantation genetic screening (PGS) in older female age group undergoing IVF-ET. Such procedures allow clinicians to transfer a single blastocyst with confidence that this policy will not reduce pregnancy and delivery chances, while reducing multiple pregnancies (Forman et al., 2013). Therefore, based on Forman et al. (2013) data PGS may extend the indication of SBT to older female age group.

We found it difficult to convince couples to participate in this study despite the fact that the cost of IVF-ET cycle was 40% less than regular fees (our study was partially supported by a grant from Ferring Pharmaceuticals). Although 353 patients fulfilled the inclusion criteria only 191 (53.8%) agreed to participate in the study. The main reason for refusal was the concern, that SBT may reduce pregnancy chances. Similarly, Gardner et al. (2004) had difficulty recruiting patients in their study (Gardner et al., 2004). Such findings highlight the importance of couples’ education of potential risks, complications, and cost of multiple pregnancies, including twin pregnancy. It also suggests that more work is needed at several levels including healthcare providers, health insurance plans and the media to provide the education and support needed for couples undergoing IVF-ET (Gerris, 2005). There are many reasons behind the decision to proceed with double embryo transfer. Studies have found that couples that opt for multiple embryo transfer have a positive attitude towards twin pregnancy, and a tendency towards failure to appreciate the health risks associated with twins (Hojgaard et al., 2007; Newton et al., 2007; Coetzee et al., 2007). In addition, to pregnancy rates, the cost effectiveness of IVF-ET is an important aspect to be considered with SET versus DET. Some studies have found that although more IVF-ET cycles may be required when using SET, the overall cost per child is the same, and sometimes cheaper than DET (Gardner et al., 2004; De Sutter et al., 2002). In situations when the patient is paying for possible additional transfers and cycles, cost remains the main hurdle to receiving SET during IVF-ET treatment. In this regard, it appears that the push for e-SET or e-SBT will work better in countries and states where there is a mandate for covering for IVF treatment (Moustafa et al., 2008). It is clear that the burden of high cost IVF-ET treatments on the community is attributed to the cost of caring for premature babies born as a result of deliveries of multiple gestations. However, infertile couple undergoing IVF-ET should not be expected to be responsible for such burden if insurance coverage does not include such treatment. Elective SET/SBT will also be more acceptable with higher awareness of the risk of multiple pregnancies and with the current high pregnancy rates of FTBT cycles (Maheshwari et al., 2012; Veleva et al., 2009). This is especially the case in view of recent data that suggest better results with FTBT compared to fresh cycles (Shapiro et al., 2011; Roque et al., 2013). Patient education about the risks of multiple gestations is paramount for informed consent and to allow for autonomy for the patient. Ryan et al. (2007) found that after education and knowledge of twin risks, a significant number of subjects opted for lower gestational number (Ryan GL et al., 2007). His study revealed that educational material can affect decisions on the number of embryos transferred. Initially in his study 20% of patients desired twins. However, after education more people preferred e-SBT than DBT. This decreased the twin pregnancy rate by 45% without affecting pregnancy rate (Ryan et al., 2007).

Interestingly and in contrast to the current medical consensus Gleicher et al. (2009) published data to suggest that contrary to the concept of e-SET, which has gained wide popularity, twin deliveries represent a favorable and cost-effective treatment outcome that should be encouraged for infertile patients who want more than one child (Gleicher and Barad, 2009). Gleicher et al. (2009) cites that twin pregnancies after IVF treatment demonstrates a 40% lower perinatal mortality than those spontaneously conceived. The data in our study support this notion as the majority of patients with twin gestation has favorable outcome. In addition, during the fresh cycles there was no significant difference in the mean gestational age, incidence of preterm birth and incidence of severe preterm birth between the two groups. Additionally, Gleicher and Barad (2009) find folly in previous cost analysis on the basis of the assessed reference outcomes, stating that the outcomes should be based on the number of newborn infants, and lifetime costs, rather than short term costs of treatments and hospital expenses (Gleicher and Barad, 2009). In a more recent study, Gleicher (2013) suggested that SET for all patients irrespective of their age group is irrational and may reduce the chances of having a child for many infertile couples (Gleicher, 2013). In addition, Gleicher et al. (2007) reported lower pregnancy rates in Europe compared to USA and he suggested that the increase use of SET could further widen this gap.

The strength of this study stems from being a randomized clinical study, with results contributing to the pool of knowledge in the quest to find the best recommendations for number of embryos/ blastocysts to be transferred. The two-studied populations were similar in regards to the majority of demographic data, ovarian stimulation characteristics, and embryology data confirming that randomization was successful. During the fresh cycles multiple Binary logistical regression analysis of the effects of the few variables (in demographic data, ovarian stimulation data and embryology data) that were found to be significantly different between the two groups showed that such differences had no impact on the clinical pregnancy rate. Other points of strength include the fact that the study was conducted at a single unit, and the fact that the majority of ET was performed by the senior author using identical techniques; thereby eliminating an important confounding factor that affects implantation rates. Another point of strength is the fact that this is the second randomized clinical trial that compared SBT to DBT, albeit it is larger than the first study by Gardner et al. (2004). The main limitation of this study was the small number of patients that eventually were randomized, which affected the statistical power. We started with a satisfactory sample size (353); unfortunately, we lost 47% of those eligible for the study, as they were not comfortable participating in the study as explained before. The same difficulty to recruit patients for a similar study was also experienced by Gardner et al. (2004) (Gardner et al., 2004). In addition, in lieu of the randomization criteria of our study, 91 patients were not suitable for randomization. Therefore, the sample size in our study may limit the generalizability of our results. However, this is a pilot study and its aim is to examine if there was clinically significant differences between the groups. The results of our study should be interpreted in that context. In addition, the only other randomized controlled trial that compared e-SBT to DBT was under powered and reported a smaller number of patients (Gardner et al., 2004). We believe that another prospective randomized controlled trial with a larger sample size, perhaps through a multi-centric study is needed to confirm our preliminary findings. While this reduction in sample size may be seen as a limitation, it highlights the need for patients’ education. As previously stated, patients’ education about the risks of multiple gestations is paramount for informed consent and to allow for autonomy of the patients (Ryan et al., 2007). The apprehension found to participate in this study due to concern of an anticipated chance of decreasing pregnancy rate with SBT shows the need for continued education.

## Conclusion

In young patients (< 35years) with favorable reproductive potential, although SBT appears to reduce clinical pregnancy and live-birth rates, excellent pregnancy outcomes can be achieved. Clinicians must weigh the benefits of DBT against the risk associated with multiple pregnancies in each specific patient before determining the number of blastocysts to be transferred. Issues to be taken into consideration before deciding on SBT versus DBT should include couples desire, legislation, cost analysis/insurance coverage, female medical conditions that can complicate twin pregnancy and availability of neonatal intensive care unit to care for premature birth. In addition, guidelines by societies such as SART in USA and the European Society of Human Reproduction and Embryology should be followed. Therefore, counseling to uphold no maleficence and autonomy of the couples pursuing the number of blastocysts to be transferred should always be exercised.
